# Unexplained Pancytopenia in a Patient with 5q35.2-q35.3 Microduplication Encompassing *NSD1*: A Case Report

**Published:** 2018-10-01

**Authors:** Sungwoo Park, Gyeong-Won Lee, Eun-Ha Koh, Hyun-Young Kim

**Affiliations:** 1 Department of Internal Medicine, Gyeongsang National University Hospital, Gyeongsang National University School of Medicine, Jinju, Republic of Korea; 2 Department of Laboratory Medicine, Gyeongsang National University Hospital, Gyeongsang National University School of Medicine, Jinju, Republic of Korea

**Keywords:** Pancytopenia, Chromosomal abnormality, Microarray

## Abstract

The 5q35.2-q35.3 duplication phenotype is characterized by growth delay, microcephaly, mental retardation and delayed bone aging. However, there has been no reports on the occurrence of pancytopenia as a consequence of 5q35.2-q35.3 duplication. A 42-year-old male visited the emergency room due to multiple trauma. He had been diagnosed with mental retardation in the past. No previous history of severe bleeding symptom was also reported. Complete blood cell counts were leukocyte 3.51×10^9^/L, neutrophil 0.19×10^9^/L, hemoglobin 8.3 g/dL, hematocrit 25.0%, and platelet 4.0×10^9^/L. There was no relevant history of any medication intake and there were no other haematological parameters leading to the persistent pancytopenia. A bone marrow biopsy revealed hypercellular marrow with increased trilineage hematopoiesis. The uptake of fluorodeoxyglucose was increased in multiple lymph nodes, bone and spleen in positron emission tomography–computed tomography. A biopsy of the right axillary lymph node was performed and histologic findings were unremarkable. The chromosomal microarray revealed a 3.46 Mb microduplication at the 5q35.2-q35.3 site including *NSD1*. The patient had distinctive features related to atypical pancytopenia. Various managements for pancytopenia had no effect on the patient. However, there were no complications such as massive bleeding or serious infection compared to the severity of pancytopenia during a follow-up of 3 months. In addition, periodic patterns of deterioration and improvement in pancytopenia appeared spontaneously. Since it is rare for these distinctive features of pancytopenia and chromosomal abnormality to coexist, it is important to investigate the association. In the current study, we describe the first case of 5q35.2-q35.3 microduplication encompassing *NSD1* with unexplained pancytopenia.

## Introduction

All 3 components of blood are reduced to below the normal reference range in pancytopenia. A variety of blood and non-hematologic diseases can have a primary or secondary effect on the bone marrow, leading to pancytopenia^1^. However, various international studies have reported few cases of pancytopenia with normal marrow ranging from 3.38% to 10.5%^2^.

Meanwhile, loss-of-function mutations of *NSD1* and 5q35 microdeletions encompassing *NSD1* are a major cause of Sotos syndrome (Sos), which is characterized by overgrowth, macrocephaly, characteristic facies and variable intellectual disability (ID)^3^. In contrast, microduplications of 5q35.2–q35.3 including *NSD1* have been reported in a few patients so far^4^. However, there have been no reports on the simultaneous occurrence of pancytopenia. Here, we report a patient with microduplication of 5q35 including *NSD1* detected by molecular karyotyping and co-occurring pancytopenia.

## Case presentation

A 42-year-old male with mental retardation visited the emergency room due to multiple trauma. The diagnosis of pancytopenia was confirmed by complete blood count test. No intake of medications and previous history of severe bleeding symptom were reported. Consciousness at the time of admission was clear, and there were no local neurological abnormalities on neurological examination. On chest examination, tenderness in the left rib was confirmed, and no specific findings on abdominal examination were observed.

Complete blood count test showed leukocyte 3.51×10^9^/L, neutrophil 0.19×10^9^/L, hemoglobin 8.3 g/dL, hematocrit 25.0%, platelet 4.0×10^9^/L. There were no abnormal cells in peripheral blood (PB) smear. Biochemical analysis showed glucose 173 mg/dL, lactate dehydrogenase 459 ng/mL, total protein 5.5 g/dL, albumin 3.5 g/dL, and total bilirubin 0.41 mg/dL. The following laboratory results were obtained: Alkaline phosphatase 64 IU/L, aspartate transaminase 34 IU/L, alanine transaminase 40 IU/L, blood urea nitrogen 9.4 mg/dL, creatinine 0.93 mg/dL. Blood coagulation test was normal. Anemia profile showed folate 1.65 ng/mL, vitamin B_12_ 294.9 pg/mL, ferritin 189.0 ng/mL, iron 45mg/dL, total iron-binding capacity (TIBC) 233 mg/dL, and transferrin saturation 19%. Antinuclear and antineutrophil cytoplasmic antibody test was negative. There was no clonality on paroxysmal nocturnal hemoglobinuria (PNH) flow cytometry. Hepatitis B and C, human immunodeficiency virus, cytomegalovirus, parvovirus were not detected in PCR, and Epstein–Barr viral immunoglobulin M (IgM) was negative. The beta-glucocerebrosidase activity for Gaucher disease and chromosomal breakage study for Fanconi anemia were normal (Table 1).

**Table 1.** Evaluation of pancytopenia

**Parameter**	**Value**
Infectious disease	
Parvo B19 virus	Negative
HIV	Negative
HBV	Negative
HCV	Negative
CMV	Negative
EBV	Negative
Immunology	
ANA	Negative
ANCA	Negative
RF	Negative
Cold agglutinin	Negative
Direct/indirect coombs	Negative
Other	
Vitamin B12 (pg/mL)	294.9
Folate (ng/mL)	1.65
PNH screen	Negative
Fanconi test	Negative
Gaucher test	Negative
Chromosomal karyotype	46, XY, t(6;9) (q21;q21)

HIV, human immunodeficiency virus; HBV, hepatitis B virus; HCV, hepatitis C virus; CMV, cytomegalovirus; EBV, Epstein-Barr virus; ANA, antinuclear antibody; ANCA, anti-neutrophil cytoplasmic antibody; RF, rheumatoid factor; PNH, paroxysmal nocturnal hemoglobinuria

**Figure 1:**
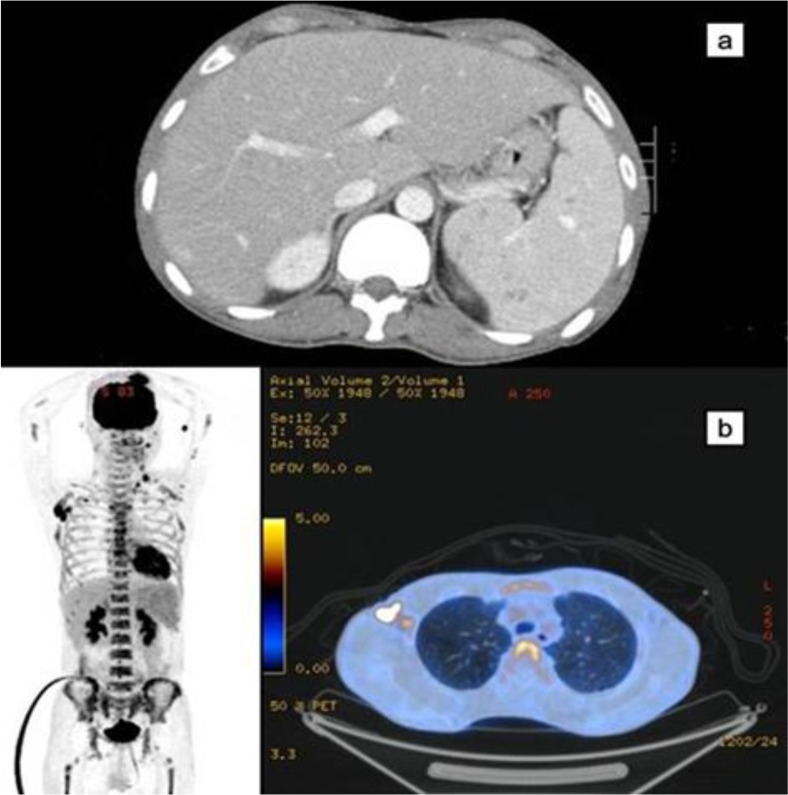
(a) Abdomen computed tomography shows newly appeared splenomegaly. (b) Positron emission tomography–computed tomography shows fluorodeoxyglucose uptake increased in conglomerated right axillary, left axillary, left supraclavicular, left and right cervical lymph nodes, along with enlarged spleen, and bones.

Brain computed tomography (CT) revealed a mild traumatic intracerebral hemorrhage and maxillary and arch orbital fracture at the time of admission. Chest CT revealed multifocal liver-like opacity with suspected bruising of the lung. During the 2-week of follow-up, the brain CT showed improvement in hemorrhage, while enlarged right axillary lymphadenopathy and spleen enlargement (13.8 cm) were newly identified with contrast attenuation on chest and abdominal CT. To evaluate the cause, the positron emission tomography -computed tomography (PET)–CT at 1 month after admission demonstrated increased fluorodeoxyglucose (FDG) uptake in multiple lymph nodes, bone and spleen (Figure 1).

**Figure 2:**
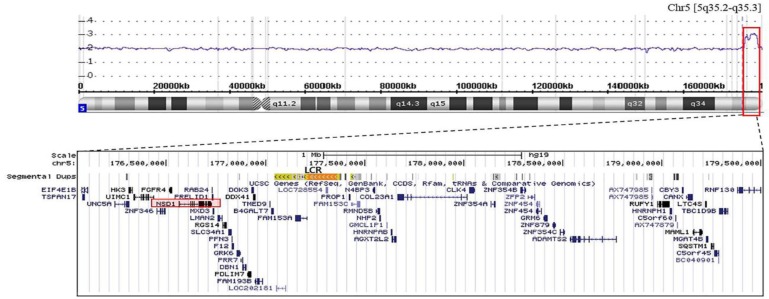
Chromosomal microarray showed a microduplication from 5q35.2 to 5q35.3(red box), arr[hg19] 5q35.2q35.3(176,062,669_179,524,425)×3, and corresponding region was displayed using the UCSC Genome Brower (http://genome.ucsc.edu/). This region included 38 OMIM genes including *FGFR4, NSD1, SLC34A1, F12, DOX41, B4GALT7, PROP1, NHP2, PHYKPL, GRM6, ADAMTS2, SQSTM1.* LCR: low-copy repeats

A lymph node biopsy was performed in right axilla, but the histologic finding showed only chronic inflammation. The bone marrow (BM) examination revealed hypercellular marrow with increased trilineal hematopoiesis (cellularity: 80–90%), and other significant findings were not found. Chromosomal study showed 46, XY,t(6;9)(q21;q21) in both of PB and BM. But the chromosomal microarray (Affymetrix CytoScan™ 750K Array) revealed a 3.46 Mb microduplication of 5q35.2-q35.3 region including *NSD1* (Figure 2).

**Figure 3:**
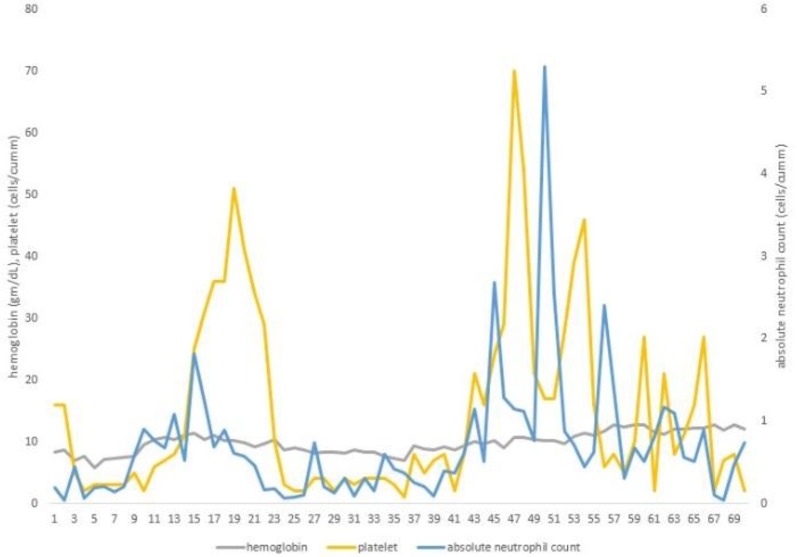
Graph showing dynamic changes in haematological parameters

Changes in hematologic parameters during the hospital stay are shown in Figure 3.

The supplement for vitamin B_12_/folic acid deficiency, intravenous immunoglobulin and continuous platelet transfusions were performed at the time of admission. One week later, granulocyte colony-stimulation factor (G-CSF) and methylprednisolone were administered for six weeks. Neutrophil and platelet counts recovered to 0.61×10^9^/L and 51.0×10^9^/L, respectively on the 20th day of admission. But on the next day, the cytopenia started to deteriorate and on the 24th day, neutrophil and platelets were 0.18×10^9^/L and 1.0×10^9^/L, respectively.

After 43 days of hospitalization, neutrophil and platelets gradually increased, and after a further 5 days, they reached 1.15×10^9^/L and 70.0×10^9^/L, respectively. However, on the next day, cytopenia started to deteriorate again and did not recover until the 70th day. After 3 months of follow-up, chest and abdominal CT showed that enlarged lymphadenopathy disappeared but the spleen remained enlarged. The patient is still under observation in the outpatient clinic with no complications related to pancytopenia.

## DISCUSSION

In the present case, the patient had distinctive features related to atypical pancytopenia. First, various managements for pancytopenia were not effective for a long period. Second, there was a fluctuating pattern of pancytopenia and no proportional relationship between platelet and neutrophil count. Third, the patient had no experience of serious infection or massive bleeding compared to severity of pancytopenia. The fever and intracerebral hemorrhage improved quickly regardless of the platelet count.

Splenomegaly occurs in a large number of hereditary diseases^5^. However, no reports described splenomegaly in patients with microduplication of 5q35.2-q35.3 heretofore. Disease states causing the splenomegaly (Fanconi disease, Gaucher disease, hematologic malignancy, hemolytic anemia and infectious disease) could be ruled out in the patient^5^. There is a rough correlation between splenic size and magnitude of thrombocytopenia^6^. However, the patient had high magnitude of thrombocytopenia compared to spleen size. These findings suggested that splenomegaly may not cause the pancytopenia in the present case. It was difficult to perform a splenic biopsy or splenectomy for further evaluation because of thrombocytopenia.

Histological examination confirmed that there was no lymphoma involvement in the axillary lymph node. In addition, the size of the lymph node decreased spontaneously on follow-up CT and the blast was not identified in bone marrow examination. Although it was difficult to differentiate cancer from inflammatory lesions in PET-CT^7^, the FDG uptake in multiple lymph nodes, bone and spleen could not be regarded as the malignancy for those reasons.

Sotos syndrome is characterized by an autosomal dominant overgrowth phenotype with dysmorphic features, advanced bone age and variable intellectual disability. The majority of affected individuals have heterozygous loss-of-function mutations within *NSD1* and 5q35 microdeletions encompassing *NSD1*. In contrast, the duplication 5q35.2.q35.3 phenotype is characterized by growth delay, microcephaly and delayed bone age in some patients, and it has been referred to as a reverse phenotype of *Sotos* syndrome^8,9^. In the present case, short stature and mental retardation of patient raised the possibility of congenital disorder. Although chromosomal study demonstrated the balanced translocation, there was no microdeletion/duplication around translocation breakpoint regions and a 3.46 Mb microduplication of 5q35.2-q35.3 site was observed. These findings were consistent with the phenotype of the reversed Stos syndrome previously reported^8^. Nicola et al. confirmed 9 genes in addition to *NSD1* with OMIM (online Mendelian inheritance in Man) annotation and compared the clinical and molecular data of all 14 patients with microduplication of 5q35.2–q35.3. Three of which were associated with disease. Each of them may contribute to the duplication phenotype although the clinical effect of a duplication of these genes is not known^8^. In the present case, 37 OMIM genes in addition to *NSD1* were identified, but there were very few reports of clinical character except for *SLC34A1* that was related to nephrolithiasis, and/or infantile hypercalcemia^10^. If molecular data from a greater number of patients with 5q35.2–q35.3 microduplication are collected, the association with pancytopenia may be confirmed.

## CONCLUSION

In present study, we reported the case of 5q35.2q35.3 microduplication including *NSD1* with pancytopenia. Since it is rare for these distinctive features of pancytopenia and chromosomal abnormality to coexist, it is necessary to perform the functional analyses for investigating the relationship between phenotype and genotype.
